# DUOX1 Silencing in Mammary Cell Alters the Response to Genotoxic Stress

**DOI:** 10.1155/2018/3570526

**Published:** 2018-04-19

**Authors:** Rodrigo S. Fortunato, Luciana R. Gomes, Veridiana Munford, Carolina Fittipaldi Pessoa, Annabel Quinet, Fabio Hecht, Gustavo S. Kajitani, Cristiane Bedran Milito, Denise P. Carvalho, Carlos Frederico Martins Menck

**Affiliations:** ^1^Laboratório de Radiobiologia Molecular, Instituto de Biofísica Carlos Chagas Filho, Universidade Federal do Rio de Janeiro, Rio de Janeiro, RJ, Brazil; ^2^Laboratório de Reparo de DNA, Departamento de Microbiologia, Instituto de Ciências Biomédicas, Universidade de São Paulo, São Paulo, SP, Brazil; ^3^Laboratório de Fisiologia Endócrina Doris Rosenthal, Instituto de Biofísica Carlos Chagas Filho, Universidade Federal do Rio de Janeiro, Rio de Janeiro, RJ, Brazil; ^4^Departamento de Patologia, Hospital Universitário Clementino Fraga Filho, Universidade Federal do Rio de Janeiro, Rio de Janeiro, RJ, Brazil

## Abstract

DUOX1 is an H_2_O_2_-generating enzyme related to a wide range of biological features, such as hormone synthesis, host defense, cellular proliferation, and fertilization. DUOX1 is frequently downregulated in lung and liver cancers, suggesting a tumor suppressor role for this enzyme. Here, we show that DUOX1 expression is decreased in breast cancer cell lines and also in breast cancers when compared to the nontumor counterpart. In order to address the role of DUOX1 in breast cells, we stably knocked down the expression of DUOX1 in nontumor mammary cells (MCF12A) with shRNA. This led to higher cell proliferation rates and decreased migration and adhesion properties, which are typical features for transformed cells. After genotoxic stress induced by doxorubicin, DUOX1-silenced cells showed reduced IL-6 and IL-8 secretion and increased apoptosis levels. Furthermore, the cell proliferation rate was higher in DUOX1-silenced cells after doxorubicin medication in comparison to control cells. In conclusion, we demonstrate here that DUOX1 is silenced in breast cancer, which seems to be involved in breast carcinogenesis.

## 1. Introduction

Cancer is the leading cause of death in economically developed countries and the second in developing countries, only behind deaths related to cardiovascular disease. In women, breast cancer is the second main cause of cancer death, exceeded only by lung cancer [1]. Breast cancer has an extensive list of risk factors associated with its development, such as age, sex, genetic predisposition, breast density, personal and familiar history of breast cancer, obesity, and early menarche [2]. Several authors suggest that a common point between various risk factors is an imbalance of redox homeostasis, which is related to the establishment and development of several tumors [3].

Reactive oxygen species (ROS), such as superoxide, hydroxyl radical, and hydrogen peroxide (H_2_O_2_), comprise a large group of oxygen-derived small molecules that include radical and nonradical species. ROS avidly interact with a large spectrum of cellular constituents, including small inorganic molecules, proteins, lipids, and nucleic acids, altering their structures and functions [4]. Many authors classify these molecules as harmful to biological organisms; however, the role of ROS has been revisited, assuming its importance in cellular redox signaling controlling several physiological mechanisms [5]. ROS can be formed as a by-product of enzyme activities, such as xanthine oxidase, cytochrome P-450, or mitochondrial electron transport chain, or directly by the NADPH oxidase (NOX) family of enzymes [6]. Unlike other oxidoreductases, NOX enzymes produce ROS in a regulated way, which is correlated to a wide range of biological features, such as hormone synthesis, host defense, cell proliferation, and fertilization. As a consequence, it is reasonable to think that any deregulation of the expression and/or activity of these enzymes can impact cellular physiology and the development of several diseases [7].

The NOX/DUOX family is composed of seven members, NOX1–NOX5 and DUOX1 and DUOX2, which are differentially expressed among tissues [8]. DUOX1 (dual oxidase 1) is present in different cell types of various tissues, but its most characterized function is in mucosal surfaces of the gastrointestinal and respiratory tracts, where it is involved in host defense [9]. Interestingly, while other NOX enzymes are upregulated in cancer cells, explaining the higher amount of ROS generated by them in comparison to their normal counterparts [10], previous studies have shown a decreased DUOX1 expression in lung and liver cancers [11, 12]. Here, we show that DUOX1 is downregulated in breast cancer and that its expression is crucial to the physiology of mammary epithelial cells, once nontumor cells silenced for DUOX1 show increased proliferation rate and decreased migration, adhesion, and cytokine secretion. Finally, the physiological alterations elicited by the downregulation of DUOX1 seem to modify the cellular responses to doxorubicin, one of the most commonly used chemotherapeutic agent for breast cancer treatment [13].

## 2. Materials and Methods

### 2.1. Chemicals, Reagents, and Cells

All chemicals and reagents were purchased from Sigma-Aldrich Co. (St. Louis, MO, USA), unless otherwise specified. Nontumor human mammary epithelial cell lineage MCF12A was maintained in phenol red-free DMEM/F12 medium containing 5% horse serum (Gibco®/Life Technologies, Carlsbad, CA, USA), penicillin and streptomycin (2%), and amphotericin B (1 mg/mL) and supplemented with cholera toxin (100 ng/mL), EGF (20 ng/mL), insulin (10 *μ*g/mL), and hydrocortisone (500 ng/mL). The MCF7 (ER-positive) and the MDA-MB-231 (triple-negative) human breast tumor cell lines (ATCC) were maintained in phenol red-free DMEM and RPMI medium, respectively, containing 10% fetal bovine serum (Gibco/Life Technologies, Carlsbad, CA, USA), penicillin, and streptomycin (2%). All cells were maintained at 37°C in an atmosphere of 5% CO_2_/95% air.

Doxorubicin (Sigma-Aldrich) was used as a genotoxic agent in some experiments. The cells were incubated with 25 nM doxorubicin in medium for 24 or 48 h. After treatment, the cells were harvested at the indicated time.

### 2.2. Tissue Samples and Immunohistochemistry

Ten women diagnosed with breast cancer were chosen for the study. Recruitment of patients was carried out in the service of Gynecology and Obstetrics of University Hospital Clementino Fraga Filho, UFRJ, and at Moncorvo Filho Institute of Gynecology, UFRJ, during hospitalization for performing surgery of patients already diagnosed with breast cancer through biopsy. Patients that have previously undergone hormone treatment and/or chemotherapy and/or neoadjuvant radiotherapy were excluded. We also excluded patients with a history of second primary tumor of any location subjected or not to previous treatment. Tumor and nontumor tissues were evaluated by a pathologist present in the operating room during surgery. Tissues were collected from surgical resection, quick frozen, and stored in liquid nitrogen immediately after excision. All histopathological findings and immunohistochemistry were performed at the Clinical Pathology Laboratory of the University Hospital Clementino Fraga Filho, UFRJ, and reviewed. Written informed consent was obtained from each participant when they enrolled in the follow-up study. All methods in this study were performed in accordance with the relevant guidelines and regulations. The project was approved by the Ethics Committee of the University Hospital Clementino Fraga Filho, UFRJ (CEP15294913.8.0000.5257).

### 2.3. Quantitative PCR (qPCR)

Total RNA was extracted using RNeasy Mini Kit (Qiagen, Venlo, Netherlands), and 2 *μ*g was used for reverse transcription with High Capacity cDNA Reverse Transcription Kit (Applied Biosystems, Foster City, CA, USA). Quantitative real-time PCR was performed with SYBR Green Master Mix (Thermo Fisher Scientific, Waltham, MS, USA). Data were analyzed using the 2^−∆∆Ct^ method. GUS was used as the housekeeping control gene. Primers used were DUOX1 forward: 5′-AGGAGTGGCATAAGTTTGAGG-3′, DUOX1 reverse 5′-GCGTCACCCAGATGAAGTAG-3′, IL-6 forward: 5′-GGCACTGGCAGAAAACAACC-3′, IL-6 reverse: 5′-GCAAGTCTCCTCATTGAATCC-3′, IL-8 forward: 5′-CTGGCCGTGGCTCTCTTG-3′, IL-8 reverse: 5′- CCTTGGCAAAACTGCACCTT-3′, GUS forward 5′-AGGTGATGGAAGAAGTGGTG-3′, GUS reverse 5′- AGGATTTGGTGTGAGCGATC-3′.

### 2.4. Cellular Proliferation and Adhesion Assay (xCELLigence)

Cells were seeded onto a 96-well E-Plate (Roche, Basel, Switzerland), with interdigitated microelectrodes at the bottom of each well, at a concentration of 3 × 10^3^ cells per well. After 30 min at room temperature, the E-Plate was placed onto an xCELLigence real-time cell analyzer (RTCA) SP system device (Roche), located inside a tissue culture incubator, where cells were left to grow. Impedance was continuously measured over time. The increase in the number and size of cells attached to the electrode sensors leads to impedance increase, from which the cell index values displayed at the plot, used for the evaluation of cell proliferation, were derived. To assess cellular adhesion, the cell index values were collected after 9 h of plating. The cell index correlates with the number of cells attached to the bottom of the well. In some experiments, 25 nM doxorubicin was added 24 h after plating and the cellular proliferation was evaluated over time.

### 2.5. Migration and Invasion Assays

Cells were plated on top of chambers of 8 *μ*m pore transwells (EMD Millipore, Billerica, MS, USA) in low-serum medium. The medium present in the bottom chamber, supplemented with 5% horse serum, was used as a chemoattractant. These cells were allowed to migrate over a period of 24 h. For the invasion assays, the chambers were previously coated with Matrigel® (Corning, Corning, NY, USA) and the cells were allowed to invade for 48 h. Cells remaining at the top chamber were removed, and those present at the bottom of the filter were stained and fixed with Coomassie Blue 0.125% in methanol: acetic acid: H_2_O (45 : 10 : 45, *v*/*v*/*v*) for 15 min. Representative images using a 10x objective lens were taken. Triplicate wells were used per condition in two independent experiments. Using the ImageJ® program, the relative cellular migration and invasion were quantified from the images obtained (10x objective lens) under each experimental condition.

### 2.6. Intracellular and Mitochondrial ROS Levels

Cells were dissociated and incubated with 10 *μ*M H_2_DCF-DA (Invitrogen®/Life Technologies) for 30 minutes or with 5 *μ*M MitoSOX Red (Invitrogen/Life Technologies) for 10 minutes at 37°C. Mean fluorescence intensity was detected by flow cytometry using Guava easyCyte (Millipore). Excitation and emission settings were 495/520 nm for H_2_DCF-DA and 510/580 nm for MitoSOX Red.

### 2.7. Extracellular H_2_O_2_ Production

Extracellular H_2_O_2_ production was quantified by the Amplex Red/HRP assay, which detects the accumulation of a fluorescent oxidized product. The cells were treated or not with 25 nM doxorubicin for 24 h. After that, 1 × 10^5^ cells in phenol red-free balanced salt solution (BSS) were incubated with D-glucose (1 mg/mL), SOD (100 U/mL; Sigma-Aldrich), HRP (0.5 U/mL; Roche), and Amplex Red (50 *μ*M; Molecular Probes, Eugene, USA) and the fluorescence was measured in a microplate reader (Victor X4; PerkinElmer, Waltham, MS, USA) for 40 min in the wavelength of 530 nm excitation and 595 nm emission. H_2_O_2_ generation (nmol H_2_O_2_ × h^−1^ × 10^5^ cells) was calculated using standard calibration curves.

### 2.8. Cytokine Secretion in Culture Supernatants

Cells were treated or not with 25 nM doxorubicin for 48 h. After that, the cell medium was collected, centrifuged at 800 ×g and stored at −80°C. After that, we performed the CBA assay using the Human Inflammatory Cytokines Kit (BD Biosciences, San Jose, CA, USA) following the manufacturer's instructions. For the acquisition of data, we used the software “BD Accuri C6” using the settings “BD Accuri CBA Template Kit.” The analysis was performed using the FCAP 3.0 software.

### 2.9. Active Caspase 3 Analysis

Cells were treated or not with 25 nM doxorubicin for 48 h. After that, they were trypsinized, washed once with PBS, and then fixed with 70% cold ethanol (*v*/*v*) for at least 24 h at −20°C. To quantify the active form of caspase 3, the immunostaining was performed at room temperature for 1 h and 30 min using a FITC-conjugated antiactive caspase 3 antibody (559341 BD Pharmingen, San Diego, CA, USA) diluted at 1/10. Samples were applied on a Guava flow cytometer (Millipore) and the data were analyzed with CytoSoft Data Acquisition and Analysis Software (Millipore).

### 2.10. Statistical Analysis

All results were expressed as mean ± standard error of the mean (SEM) and were analyzed using one-way ANOVA followed by Bonferroni's multiple comparison test, except those related to doxorubicin treatment in which two-way ANOVA was utilized. Statistical analyses were performed using GraphPad Prism software (version 5.01, GraphPad Software Inc., San Diego, USA). *P* < 0.05 was considered statistically significant.

## 3. Results

### 3.1. DUOX1 Expression Is Downregulated in Tumor Tissues and Tumor Cell Lineages

**Figure 1:**
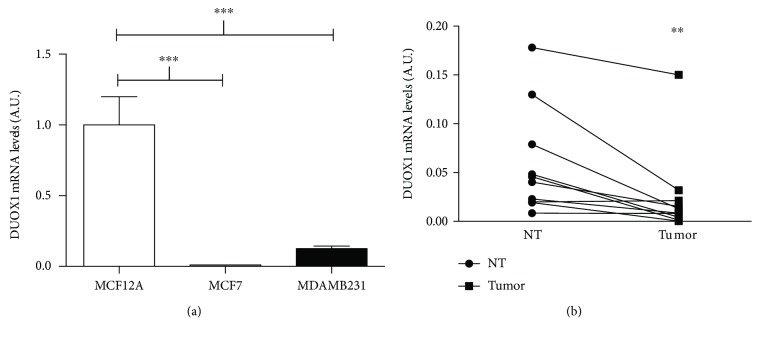
DUOX1 expression is downregulated in breast cancer cell lines and tissues. DUOX1 mRNA levels were evaluated by quantitative RT-PCR in breast cancer cell lines (a) and in paired cancerous and adjacent breast tissue samples (b). Data are expressed as fold change relative to MCF12A for breast cancer cell line comparison and relative to the respective control to breast tissue samples. Results are expressed as mean ± SEM of three independent experiments. ^∗∗^*P* < 0.01; ^∗∗∗^*P* < 0.001.

Previous studies have shown a decreased DUOX1 expression in lung and liver cancers [11, 12]. As DUOX1 is expressed in mammary nontumor cells, we decided to compare DUOX1 expression between nontumor and tumor breast cell lines and human breast tissues. As shown in Figure 1(a), tumor cells (MCF7 and MDA-MB-231) have less DUOX1 mRNA levels than nontumor cells, MCF12A. Strikingly, we could not detect any DUOX1 mRNA expression in the MCF7 cell line. Importantly, when comparing tumor breast tissue samples with nontumor samples from the same patient, we observed that tumor samples expressed significantly less DUOX1 mRNA levels (Figure 1(b)). Interestingly, the histopathological and immunohistochemical characterization of human breast cancer samples shows that they are very heterogeneous in relation to its expression of ER, PR, and HER-2 receptors, as well as proliferation rates (Table 1).

### 3.2. Effect of DUOX1 Depletion on Cell Proliferation, Adhesion, Migration, and Invasion

**Figure 2:**
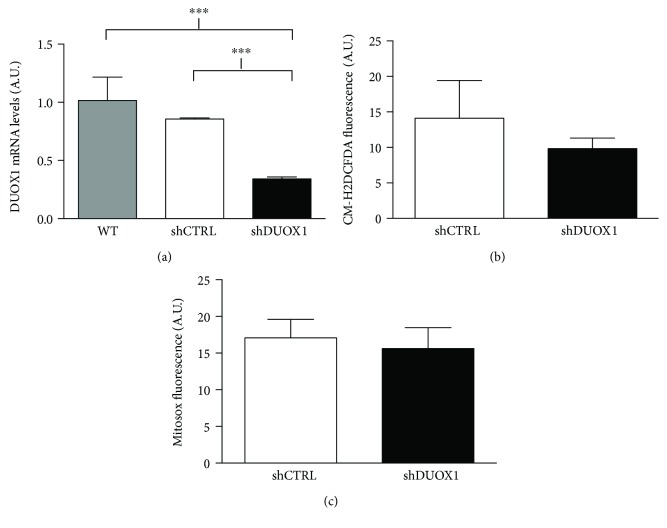
Characterization of DUOX1-silenced cell. Nontumor MCF12A cells were silenced for DUOX1 by shRNA (shDUOX1) and compared to the scramble control (shCTRL). DUOX1 mRNA levels were evaluated by quantitative RT-PCR (a), while intracellular reactive oxygen species (ROS) levels (b) and mitochondrial ROS production (c) were evaluated by flow cytometry utilizing the CM-H2DCF-DA and MitoSOX probes, respectively. Results are expressed as mean ± SEM of two independent experiments. ^∗∗∗^*P* < 0.001.

**Figure 3:**
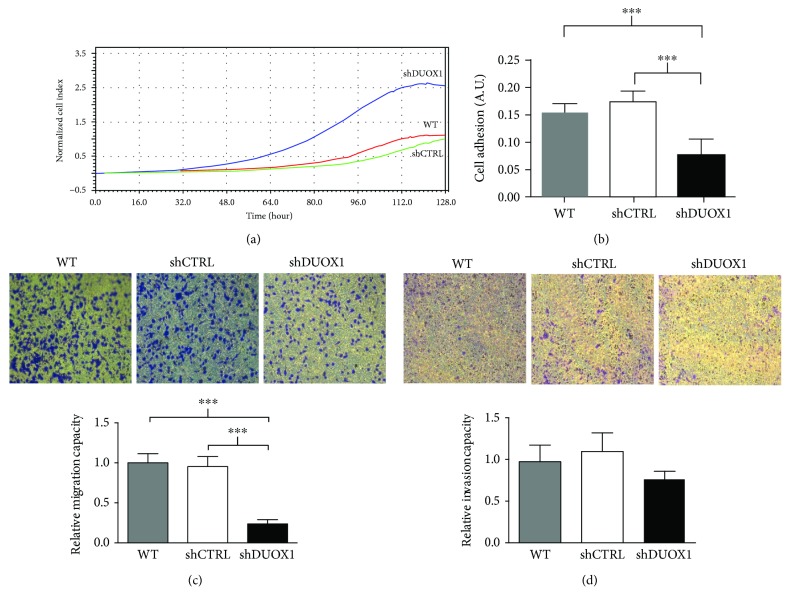
DUOX1 silencing increases proliferation and decreases adhesion and migration in nontumor MCF12A cells. Cellular proliferation (a) and adhesion (b) were evaluated by the xCELLigence system in DUOX1-deficient (shDUOX1) and control (shCTRL) cells. For migration (c) and invasion (d), the cells were allowed to migrate through uncoated transwells for 8 h or invade through Matrigel-coated transwells for 24 h. The number of cells at the bottom of the transwell filters was counted at the end of each assay. Results are presented as means ± standard errors from three independent experiments. ^∗∗∗^*P* < 0.001.

To understand the role of DUOX1 in breast tumorigenesis, we downregulated the expression of DUOX1 in the nontumor cell line MCF12A, using a specific shRNA sequence (shDUOX1). As shown in Figure 2(a), there was a significant decrease of DUOX1 expression in the MCF12A shDUOX1 cell line, when compared to MCF12A cells transduced with the shRNA scramble control sequence (shCTRL). However, there were no significant differences neither in the levels of intracellular ROS (Figure 2(b)) nor in mitochondrial ROS (Figure 2(c)) between shDUOX1 and shCTRL cells. On the other hand, cellular proliferation was higher in shDUOX1 cells in comparison to its control (Figure 3(a)), while cellular adhesion (Figure 3(b)) and migration (Figure 3(c)) were lower. No differences were observed in cellular invasion capacity (Figure 3(d)).

Three-dimensional (3D) epithelial culture model is a useful approach to understand histological abnormalities found in breast cancer, once the tissue architecture can be recapitulated in 3D culture, but not in 2D [14]. Thus, shDUOX1 cells and their controls were cultivated in 3D for 10 days, but no changes in their morphological phenotypes were observed (data not shown).

### 3.3. DUOX1 Downregulation Decreases Extracellular H_2_O_2_ Production and IL-6 and IL-8 Expression and Secretion after Genotoxic Stress

**Figure 4:**
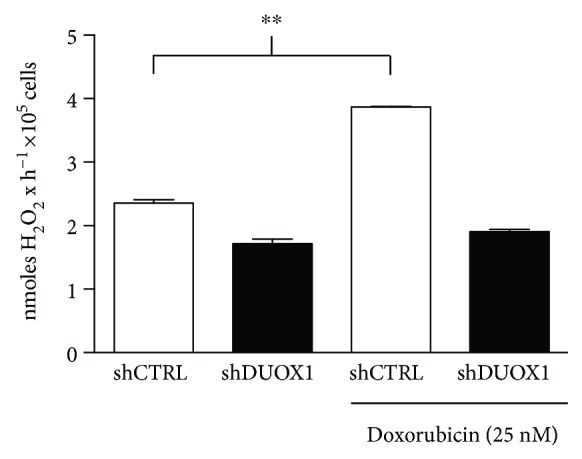
Extracellular H_2_O_2_ generation is increased after genotoxic stress. DUOX1-deficient (shDUOX1) and control (shCTRL) cells were treated or not with 25 nM doxorubicin for 24 h. Extracellular H_2_O_2_ generation was evaluated by Amplex Red/HRP assay. Results are expressed as mean ± SEM of three independent experiments. ^∗∗^*P* < 0.01.

It was shown that DUOX1 is related to the increased secretion of IL-6 and IL-8 [15, 16]. Interestingly, these cytokines are secreted by cells in response to stressful stimuli, including genotoxic stress. In damaged cells, IL-6 and IL-8 are involved in cell growth inhibition and enhancement of DNA damage response [17, 18]. As shown in Figure 4, doxorubicin treatment increased extracellular H_2_O_2_ production in shCTRL cells, but this response was blunted in shDUOX1 cells.

**Figure 5:**
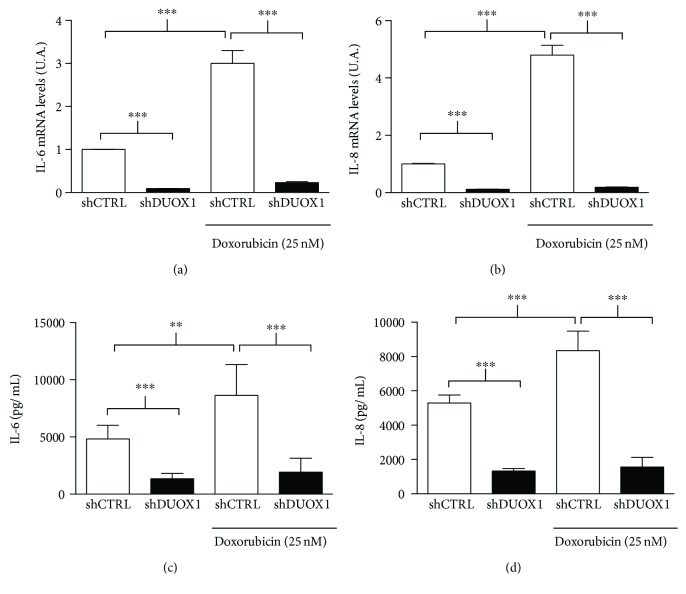
DUOX1 silencing blunts IL-6 and IL-8 mRNA levels and secretion increase after genotoxic stress. DUOX1-deficient (shDUOX1) and control (shCTRL) cells were treated or not with 25 nM doxorubicin for 48 h. IL-6 (a) and IL-8 (b) mRNA levels were evaluated by quantitative RT-PCR; IL-6 (c) and IL-8 (d) secretion was measured by flow cytometry utilizing the CBA assay. Results are expressed as mean ± SEM of three independent experiments. ^∗∗∗^*P* < 0.001.

Interestingly, we observed lower basal levels of IL-6 and IL-8 concentrations and mRNA expression in the culture medium of shDUOX1 cells compared to control cells (Figure 5). Doxorubicin treatment increased IL-6 and IL-8 gene expression and secretion in the control cells, but no stimulatory effect was observed in shDUOX1 cells, suggesting that DUOX1 may be involved in IL-6 and IL-8 secretion induced by genotoxic stress (Figures 5(a)–5(d)).

### 3.4. DUOX1 Depletion Alters Doxorubicin-Induced Cytotoxicity

**Figure 6:**
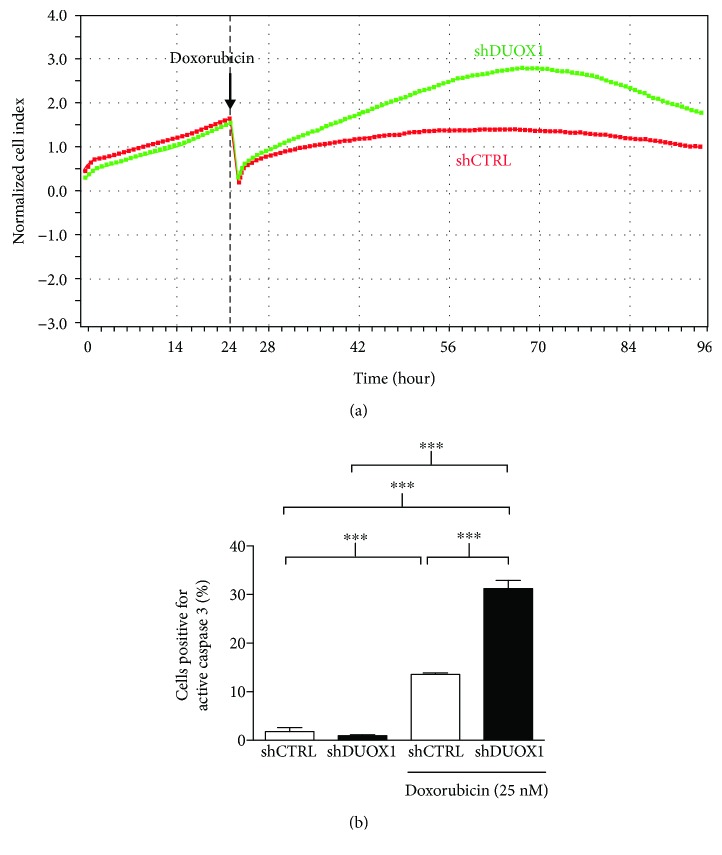
DUOX1 silencing alters genotoxic stress cellular responses. Real-time curves of cellular proliferation were obtained using the xCELLigence system for DUOX1-deficient (shDUOX1) and control (shCTRL) cells pretreated with 25 nM doxorubicin, for 24 h (arrows) (a). The active form of caspase 3 was quantified by flow cytometry, 48 h after doxorubicin treatment (b). Results are expressed as mean ± SEM of at least two independent experiments. ^∗∗∗^*P* < 0.001.

As shown in Figure 6(a), after doxorubicin treatment, the proliferation rate was higher in shDUOX1 cells when compared to the control cells. To gain further insight into the mechanisms related to the differences observed in doxorubicin-treated cells, active caspase 3-positive cells were quantified by flow cytometry, indicating cellular death by apoptosis (Figure 6(b)). As expected, doxorubicin treatment significantly increased active caspase 3-positive cell levels in both cell lines; however, we observed a more pronounced effect in the shDUOX1 cell line than the shCTRL cell line.

## 4. Discussion

Tumor cells are generally subjected to higher levels of ROS than the corresponding normal cells [3, 19, 20]. It has been suggested that the greater ROS generation observed in tumor cells may be attributed, at least in part, to an increased expression of NADPH oxidase enzymes, as observed in tumor tissues and tumor cell lines of various types [21–23]. Interestingly, we observed a lower DUOX1 expression in breast tumor cells and tumor tissues in comparison to its control. The levels of DUOX1 expression does not seem to correlate with tumor prognosis, because the triple negative cell line MDA-MB-231 presented the same levels of DUOX1 mRNA than the ER-positive breast tumor cell MCF7. Moreover, the histopathological and immunohistochemical characterization of the tumor samples shows that they are very heterogeneous in relation to the expression of hormone receptors and proliferation, but nevertheless, DUOX1 mRNA was lower in almost all of them, when compared to their controls.

DUOX1 is present in different cellular types of various tissues, but its most characterized function is in mucosal surfaces of the gastrointestinal and respiratory tracts, where it is involved in host defense [9]. Interestingly, there is no data in the literature concerning DUOX1 function in breast epithelial cells. In order to understand the cause-effect relationship between DUOX1 loss and breast tumorigenesis, we decreased DUOX1 levels in the mammary nontumor cell line MCF12A using the shRNA approach. MCF12A shDUOX1 cell line presented a higher proliferative rate and decreased adhesion, which suggests that DUOX1 may exert a tumor suppressor role. DUOX1 loss has been reported in other tumor types, where it was also related to increased cellular proliferation, decreased migration in human lung cancer, and increased proliferation in human hepatocellular carcinoma [11, 12]. Interestingly, it has been shown in lung cancer cell lines that DUOX1 expression seems to be highly associated to the loss of E-cadherin. Moreover, DUOX1 silencing in lung epithelial and cancer cell lines was associated to epithelial-to-mesenchymal transition, which is linked to metastasis [24], and transient overexpression of DUOXA1, a maturation factor of DUOX1, in breast cancer cells affected cell-cell adhesion [25]. Our data suggest that in epithelial mammary cells, the loss of DUOX1 is not associated to an invasive phenotype, since shDUOX1 cells presented no alterations in invasion and in morphogenetic phenotype in 3D culture. Several studies have shown the role of DUOX1 in wound healing [26–29], in which H_2_O_2_ produced by DUOX1 acts as a signaling molecule, modulating adhesion and migration in epithelial regeneration after injury. In the present study, we observed a decreased migration and adhesion capacity after DUOX1 silencing. Thus, we hypothesize that this enzyme could be involved in mammary epithelial regeneration, as previously reported in other cell types.

Several studies in airway cells have shown that extracellular H_2_O_2_ production mediated by DUOX1 is related to IL-6 and IL-8 secretion [15, 16]. Interestingly, these cytokines are secreted by cells in response to various stressful stimuli, resulting in cell growth inhibition, increased ROS production, enhancement of DNA damage response (DDR), and attraction of immune system cells, acting as a potent tumor suppressor mechanism by elimination of the damaged cell [17, 18]. In addition, Ameziane-El-Hassani and collaborators [30] recently showed that DUOX1 is upregulated in human thyrocytes and in thyroid tumors after genotoxic stress induced by *γ*-ray irradiation. Moreover, these authors demonstrated that H_2_O_2_ produced by DUOX1 after irradiation is involved in the generation of DNA strand breaks and DNA damage response [30]. Based on this, we hypothesized that in normal condition, DUOX1 could be involved in IL-6 and IL-8 secretion after genotoxic stress in mammary epithelial cells; however, the physiological relevance of the lower DUOX1 expression found in breast tumor remains to be elucidated. After doxorubicin-induced genotoxic stress, extracellular H_2_O_2_ generation significantly increased in control cells, but not in DUOX1-silenced cells. Moreover, shDUOX1 cells presented lower levels of mRNA and lower secretion of IL-6 and IL-8 in basal condition when compared to control cells. Thus, our results suggest that the secretion of these cytokines is under DUOX1 control in mammary epithelial cell models. To our knowledge, this is the first data indicating such control.

Anthracycline doxorubicin is one of the most effective chemotherapeutic agent available for breast cancer treatment [13]. The mechanisms of action of this drug are based on its ability to interact with topoisomerase II and intercalate into DNA and free radical formation [31]. Doxorubicin treatment increased extracellular H_2_O_2_ generation and IL-6 and IL-8 mRNA secretion, only in control cells, with no evident effect on shDUOX1 cells. This result prompted us to investigate doxorubicin-induced cell death in our study model. shDUOX1 cells presented higher apoptosis levels after doxorubicin treatment. shDUOX1 had higher proliferation rates in comparison to its control, which led to a more pronounced effect of doxorubicin in relation to DNA damage and, consequently, cell death.

In summary, we demonstrate for the first time that DUOX1 is downregulated in breast cancer. The downregulation of DUOX1 in MCF12A cells is accompanied by higher cell proliferation rate and decreased adhesion and migration. Finally, DUOX1 enzyme seems to be involved in the cellular response against genotoxic stress induced by doxorubicin, in which the lack of this enzyme seems to blunt IL-6 and IL-8 secretion after drug exposure. Thus, our novel findings might contribute to the understanding of the biology of cancers that have decreased DUOX1 expression, which could be useful to the development of future therapeutic approaches.

## Figures and Tables

**Table 1:** Histopathological and immunohistochemical report of breast tumors.

Patients	Histopathological report	ER (%)	PR (%)	HER-2	KI-67 (%)
1	Invasive ductal carcinoma Grade III	Negative	Negative	Negative	10–20

2	Invasive ductal carcinoma Grade III	Negative	Negative	Negative	10

3	Ductal carcinoma in situ	>90	20–30	Inconclusive (2+)	<5

4	Infiltrating mucinous carcinoma	90–95	90–95	—	1–5

5	Invasive ductal carcinoma Grade III	80–90	80–90	Negative	10–15

6	Invasive ductal carcinoma Grade II	100	90–95	Negative	1

7	Invasive ductal carcinoma Grade II	90–95	3–5	Positive	15–20

8	Invasive ductal carcinoma Grade III	10	Negative	Negative	60

9	Ductal carcinoma in situ	80–90	70–80	Inconclusive (2+)	5

10	Ductal carcinoma in situ	5	5	Positive	5

ER: estrogen receptor; PR: progesterone receptor; HER-2: human epidermal growth factor receptor 2; KI-67: cellular proliferation index.
